# DEMATEL-based gamified instructional design for music teacher education

**DOI:** 10.3389/fpsyg.2026.1865021

**Published:** 2026-09-03

**Authors:** Cheng Chen, Rabiatul-adawiah Binti Ahmad Rashid, Lili Xue

**Affiliations:** 1 School of Music and Dance, Zhongyuan University of Science and Technology, Xuchang, China; 2 School of Educational Studies, Universiti Sains Malaysia, Penang, Malaysia; 3 School of Music and Dance, Zhengzhou Normal University, Zhengzhou, China

**Keywords:** academic engagement, DEMATEL, gamification, pre-service music teachers, teaching self-efficacy

## Abstract

**Background:**

Gamification has received growing attention as a strategy to enhance learning experiences and educational outcomes in higher education. However, limited evidence exists regarding the relative importance of gamification design elements and their causal relationships, particularly within teacher education contexts.

**Objectives:**

This study aimed to identify the most influential gamification design elements using the Decision-Making Trial and Evaluation Laboratory (DEMATEL) method and to examine the effects of a DEMATEL-informed gamified instructional design on academic engagement and teaching self-efficacy among pre-service music teachers.

**Methods:**

A mixed-methods approach was employed. An expert panel comprising 32 specialists in music education, educational technology, and gamification evaluated nine gamification design elements through pairwise comparisons. DEMATEL analysis was conducted to determine the prominence and causal relationships among the elements. The identified priority elements were subsequently incorporated into an eight-week gamified instructional intervention involving 78 pre-service music teachers. Academic engagement and teaching self-efficacy were assessed using pretest and posttest measures.

**Results:**

The results indicated substantial agreement among experts (Kendall’s *W* = 0.71, *p* < 0.001). Progress Tracking emerged as the most influential gamification element (D + R = 10.12), followed by Feedback (D + R = 9.94) and Goals and Missions (D + R = 9.58). Feedback exhibited the strongest causal influence within the system (D − R = 1.30), whereas Social Recognition demonstrated the highest level of dependence (D − R = −1.07). Significant improvements were observed in academic engagement, which increased from 3.42 to 4.11, *t*(77) = 10.84, *p* < 0.001, and teaching self-efficacy, which increased from 3.51 to 4.19, *t*(77) = 11.37, *p* < 0.001. Large effect sizes were observed for academic engagement (Cohen’s dz. = 1.23) and teaching self-efficacy (Cohen’s dz. = 1.29).

**Conclusion:**

The findings demonstrate that DEMATEL is an effective approach for identifying and prioritizing influential gamification design elements. The results further indicate that instructional designs emphasizing progress tracking, feedback, and goal-oriented learning activities can substantially enhance academic engagement and teaching self-efficacy among pre-service music teachers. The study provides an evidence-based framework for the design and implementation of gamified learning environments in teacher education.

## 1 Introduction

The rapid development of educational technologies has transformed the design and delivery of learning experiences in higher education (Bonfield et al., 2020; Li et al., 2021; Triantafyllou et al., 2024). Universities are increasingly expected to create learning environments that promote active participation, motivation, and meaningful engagement while simultaneously preparing students for complex professional contexts (Cao et al., 2025; Rashed et al., 2025). Within this landscape, gamification has emerged as a widely adopted instructional approach that incorporates game design elements into non-game educational settings to enhance learner motivation, participation, and achievement (Sun et al., 2025; Yu et al., 2023). Recent evidence indicates that gamification contributes positively to learning outcomes, academic performance, and student engagement across diverse educational contexts (Kokarida et al., 2026; Papadakis et al., 2026).

Gamification refers to the integration of game elements such as goals, challenges, feedback, rewards, progress indicators, and social interaction into learning environments (Li et al., 2026; Liu et al., 2025; Triantafyllou and Sapounidis, 2026). Unlike game-based learning, which relies on full-fledged educational games, gamification embeds selected game mechanics into existing instructional activities to increase learner engagement and enhance the learning experience (Chu et al., 2025; Kaißer et al., 2025). Recent meta-analyses have demonstrated that gamified learning environments produce positive effects on cognitive, affective, and skill-based learning outcomes, particularly when multiple game elements are strategically combined rather than implemented in isolation (Tu et al., 2026; Xu et al., 2025). Furthermore, recent reviews have highlighted the growing importance of gamification as a pedagogical strategy that supports engagement, motivation, persistence, and academic achievement in higher education (Keykha et al., 2026; Novkovic et al., 2026).

Among the educational outcomes associated with successful learning experiences, academic engagement occupies a central position (Huang et al., 2025; Zhang et al., 2025). Academic engagement reflects the extent to which students invest behavioral, emotional, and cognitive effort in learning activities (Wang et al., 2026). High levels of engagement are associated with improved academic performance, persistence, satisfaction, and knowledge acquisition (Faro et al., 2025; James et al., 2025). Recent systematic reviews have emphasized that engagement remains one of the strongest predictors of educational success in higher education environments (Chen et al., 2025; Prananto et al., 2025; Rizwan et al., 2025). Gamification has frequently been proposed as a mechanism for strengthening engagement because game elements create opportunities for participation, challenge, feedback, and achievement. Empirical studies have reported that gamified learning environments increase participation rates, motivation, collaboration, and sustained involvement in learning activities (Akgül and Güler, 2025; Slamet and Meng, 2025).

In addition to engagement, teaching self-efficacy represents a critical outcome within teacher education programs. Teaching self-efficacy refers to teachers’ beliefs regarding their capability to organize, manage, and implement effective instructional practices. Strong self-efficacy beliefs influence instructional decision-making, classroom management, persistence, professional growth, and student outcomes (Shi et al., 2025; Täschner et al., 2025). For pre-service teachers, developing teaching self-efficacy is particularly important because it shapes future teaching practices and professional confidence. Previous research has shown that authentic learning experiences, feedback, reflection, and collaborative activities contribute significantly to the development of teaching self-efficacy (Dong and Gedvilienė, 2025; Hikmat and Hidayat, 2026). Gamified learning environments may support these processes by providing structured opportunities for performance monitoring, goal achievement, peer interaction, and continuous feedback. Recent studies have further highlighted the growing relevance of gamification within teacher education and professional learning contexts (Heaysman and Avidov-Ungar, 2026; Rogoz and Seghedin, 2025).

### 1.1 Literature review

Gamification has become one of the most widely adopted instructional approaches in higher education due to its potential to increase learner motivation, participation, and academic achievement. Defined as the application of game design elements in non-game contexts, gamification seeks to create engaging learning experiences through mechanisms such as goals, challenges, feedback, rewards, progress tracking, collaboration, and leaderboards (Deterding et al., 2011; Kapp, 2012). Recent systematic reviews and meta-analyses have reported that gamification positively influences learning outcomes, motivation, engagement, and academic performance across diverse educational settings (Li et al., 2023; Pelizzari, 2023; Zeybek and Saygı, 2024).

The growing interest in gamification is largely attributed to its capacity to address persistent challenges in higher education, including low participation, declining motivation, and limited engagement in learning activities. Recent evidence indicates that gamification contributes to improved learner involvement, participation, and academic achievement, particularly when multiple game elements are strategically integrated into instructional design (Lampropoulos and Kinshuk, 2024; Pelizzari, 2023). However, previous studies have also emphasized that selecting the most appropriate combination of gamification elements remains a significant challenge because individual elements may produce different outcomes across educational contexts (Lampropoulos and Kinshuk, 2024; Li C. et al., 2024).

Academic engagement has emerged as one of the most frequently investigated outcomes of gamification. Engagement is commonly conceptualized as a multidimensional construct encompassing behavioral, emotional, and cognitive investment in learning activities (Fredricks et al., 2004). Previous studies have consistently demonstrated positive associations between gamification and student engagement (Li et al., 2023). A recent systematic review conducted by Ruiz et al. (2024) reported that gamification enhances participation, persistence, motivation, and involvement in educational activities. Nevertheless, the authors emphasized that engagement should be examined from a multidimensional perspective rather than being restricted to participation and motivation alone. Similarly, studies conducted in technology-enhanced learning environments have shown that gamified activities promote collaboration, knowledge construction, and active participation among learners (Moon et al., 2024).

Research has also highlighted the importance of specific gamification elements in supporting educational outcomes. Feedback, progress indicators, challenges, and goal-oriented activities have repeatedly been identified as influential components of successful gamified learning environments (David and Weinstein, 2023a). Meta-analytic evidence suggests that gamification strengthens intrinsic motivation and enhances perceptions of autonomy and relatedness, both of which contribute to sustained learner engagement (Li et al., 2023). Furthermore, recent studies have demonstrated that progress monitoring and continuous feedback support self-regulated learning, increase learner persistence, and improve academic performance (Luo, 2024).

Despite these positive findings, the effectiveness of gamification remains inconsistent across studies. While some investigations report substantial improvements in motivation and performance, others identify only moderate or context-dependent effects (Li et al., 2023). Recent reviews suggest that these inconsistencies may result from differences in instructional design, learner characteristics, educational contexts, and the selection of gamification elements (Svanberg and Bergh, 2023). Researchers have argued that the effectiveness of gamification depends not only on individual game elements but also on the interactions among those elements within the overall instructional system (Li et al., 2023).

Another limitation of the current literature is the lack of systematic approaches to prioritizing gamification design elements. Most studies investigate individual elements such as rewards, badges, or leaderboards without examining their relative importance or causal relationships (Li C. et al., 2024). Consequently, instructional designers often lack empirical guidance regarding which elements should receive priority during the design process. Although gamification research has expanded substantially over the past decade, limited evidence exists regarding the interdependencies among gamification elements and their collective influence on educational outcomes (Khaldi et al., 2023).

The Decision-Making Trial and Evaluation Laboratory (DEMATEL) method is a suitable approach to addressing this limitation, as it enables the identification of causal relationships among complex, interdependent factors. By distinguishing between cause-and-effect factors, DEMATEL facilitates the prioritization of influential elements within a system and supports evidence-based decision-making (Font-Cot et al., 2025). Despite its extensive use in management, engineering, and technology research, its use in gamified instructional design remains limited, particularly in teacher education contexts.

Therefore, a significant gap exists in the literature on identifying influential gamification design elements and integrating them into instructional interventions to improve both academic engagement and teaching self-efficacy. The present study addresses this gap by employing DEMATEL to identify and prioritize gamification design elements and subsequently utilizing these findings to develop and evaluate a gamified instructional design for pre-service music teachers.

Although previous studies have consistently reported positive effects of gamification on learner motivation, engagement, participation, and academic performance, several limitations remain evident in the existing literature. First, most investigations have focused on evaluating the effectiveness of gamification as a whole rather than examining the relative importance of individual gamification design elements. Consequently, limited evidence exists regarding which elements exert the greatest influence on educational outcomes and should therefore receive priority during instructional design. Existing research has largely concentrated on isolated components such as rewards, badges, points, or leaderboards, often without considering the complex interactions among multiple gamification elements.

Second, the causal relationships among gamification design elements remain insufficiently explored. Educational gamification studies frequently adopt predefined frameworks and evaluate learning outcomes without investigating how individual elements influence one another within the overall system. As a result, instructional designers often lack empirical guidance regarding the selection and prioritization of gamification mechanisms capable of maximizing educational effectiveness.

Third, despite the growing application of gamification in higher education, relatively limited research has examined its implementation within music teacher education. Furthermore, most previous studies have emphasized engagement, motivation, or academic performance, whereas comparatively little attention has been directed toward teaching self-efficacy as a key professional outcome. Given the importance of self-efficacy in shaping future teaching practices, additional research is needed to explore how gamified learning environments contribute to the development of professional confidence among pre-service teachers.

Finally, although the Decision-Making Trial and Evaluation Laboratory (DEMATEL) method has demonstrated considerable value in analyzing complex systems and identifying causal relationships among interrelated factors, its application to gamified instructional design remains limited. In particular, few studies have employed DEMATEL to identify influential gamification design elements and subsequently use those findings to guide the development of instructional interventions.

To address these gaps, the present study integrates DEMATEL with gamified instructional design in the context of music teacher education. The study not only identifies and prioritizes influential gamification elements but also evaluates the educational effectiveness of a DEMATEL-informed intervention. Accordingly, the study was guided by the following research objectives:

*RO1:* To identify and prioritize the most influential gamification design elements using the DEMATEL method.

*RO2:* To determine the causal relationships among gamification design elements and classify them into cause-and-effect groups.

*RO3:* To develop a DEMATEL-informed gamified instructional design for pre-service music teachers.

*RO4:* To examine the effect of the DEMATEL-informed gamified instructional design on academic engagement.

*RO5:* To examine the effect of the DEMATEL-informed gamified instructional design on teaching self-efficacy.

## 2 Materials and methods

### 2.1 Research design

This study employed a sequential mixed methods design that integrated decision modeling with instructional intervention and outcome evaluation. The research was conducted in three consecutive phases. In Phase 1, the Decision-Making Trial and Evaluation Laboratory (DEMATEL) method was used to identify, prioritize, and map the causal relationships among key gamification design elements considered relevant to music teacher education. Expert judgments were collected to determine the relative importance and influence of each design element, thereby establishing a structured basis for instructional design decisions (Falatoonitoosi et al., 2013).

In Phase 2, the findings obtained from the DEMATEL analysis were translated into a gamified instructional design. The prioritized design elements were operationalized as course activities, learning tasks, feedback mechanisms, and engagement strategies suitable for pre-service music teachers. The intervention was developed to align with the instructional objectives of the music teacher education course while maintaining feasibility within a regular university teaching schedule.

In Phase 3, the instructional design was implemented and evaluated using a single-group pretest–posttest design. Academic engagement and teaching self-efficacy were measured before and after the intervention to examine changes in participants’ self-reported learning experiences and perceived teaching capabilities. The outcome evaluation results were subsequently interpreted alongside the DEMATEL findings to determine whether the prioritized design elements could be effectively translated into practice.

**Figure 1:**
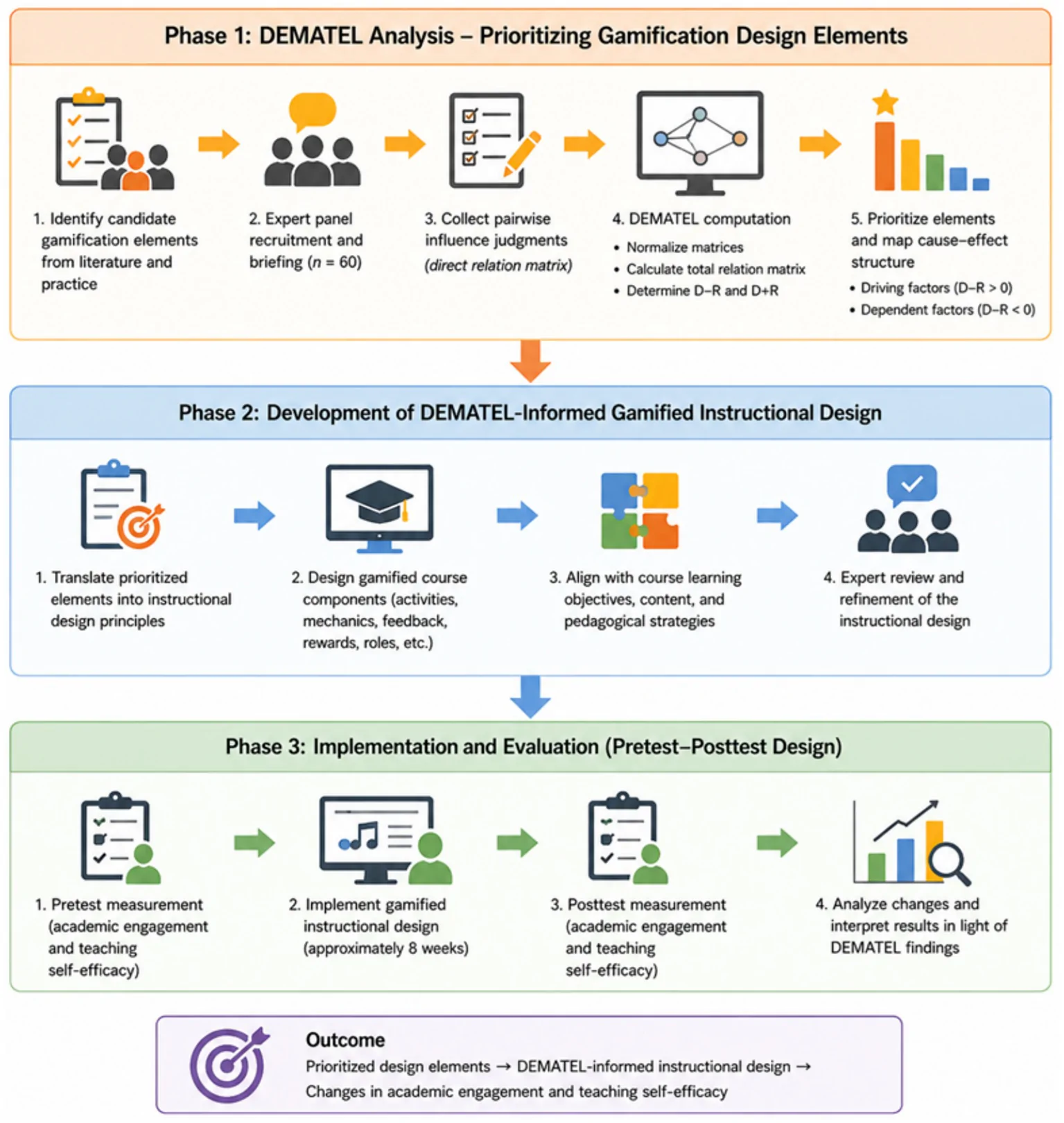
Research design of the study, showing the three phases: DEMATEL analysis, development of the gamified instructional design, and pretest–posttest evaluation.

The overall research process is illustrated in Figure 1. As shown, the study followed a systematic progression from the identification and prioritization of gamification design elements to the development of a DEMATEL-informed instructional intervention and finally to the evaluation of changes in academic engagement and teaching self-efficacy among pre-service music teachers.

### 2.2 Study context and participants

**Figure 2:**
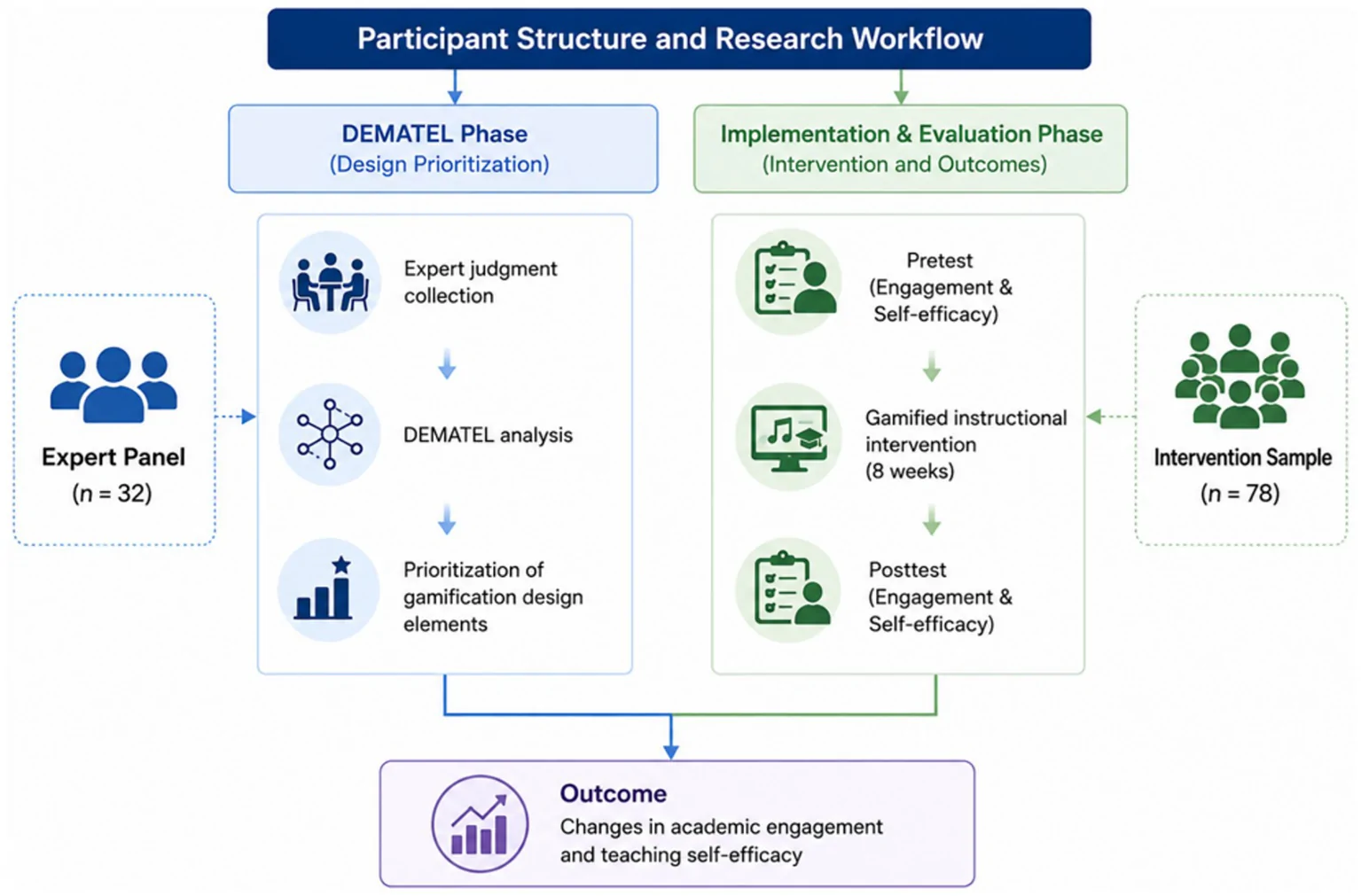
Participant structure and workflow of the study, showing the DEMATEL expert panel (*n* = 32) and the intervention sample (*n* = 78) across the design, implementation, and evaluation phases.

The study was conducted in a music teacher education program at a higher education institution in China. Two independent groups of participants were recruited to support different phases of the research. The first group participated in the DEMATEL phase and provided expert judgments for prioritizing gamification design elements. The second group participated in the instructional intervention and outcome evaluation phases. The use of separate samples ensured that the instructional design process remained independent from the subsequent implementation and assessment stages. Details of the two participant groups are presented in Sections 2.2.1 and 2.2.2. The participant allocation and research workflow are shown in Figure 2.

#### 2.2.1 DEMATEL expert panel

A purposive sample of 32 higher education teachers was recruited to participate in the DEMATEL analysis. To ensure the quality of expert judgments, participants were required to satisfy at least two of the following criteria: (a) a minimum of 5 years of university teaching experience, (b) experience in curriculum design or teacher education, (c) experience in educational technology or gamified learning implementation, and (d) scholarly publications related to teaching and learning in higher education. The panel included specialists from music education, teacher education, curriculum studies, and educational technology. This diversity was intended to provide multiple perspectives on the design and implementation of gamified learning environments. The expert panel was involved exclusively in the DEMATEL phase and did not participate in the intervention or outcome evaluation stages.

#### 2.2.2 Intervention sample

The intervention sample consisted of 78 pre-service music teachers enrolled in a music teacher education program. Participants were recruited from courses related to music pedagogy and instructional practice. Eligibility criteria included enrolment in the teacher preparation program and attendance throughout the intervention period. Participation was voluntary, and informed consent was obtained before data collection. The intervention sample was involved only in the implementation and evaluation phases of the study and did not contribute to the DEMATEL analysis. Academic engagement and teaching self-efficacy data were collected from this group using pretest and posttest assessments.

### 2.3 Identification of gamification design elements

The gamification design elements evaluated in this study were identified through a systematic review of the literature on gamification, educational technology, and teacher education. The review focused on studies examining the use of gamification in higher education and teacher preparation programs, with particular attention to design elements related to learner engagement, motivation, participation, and self-regulated learning. The objective of this stage was to establish a theoretically grounded set of design elements suitable for subsequent prioritization through the DEMATEL method.

An initial list of gamification elements was compiled from previous empirical studies, review articles, and conceptual frameworks. To improve contextual relevance, the preliminary list was examined by specialists in music education, teacher education, and educational technology. During this process, overlapping elements were consolidated, ambiguous elements were refined, and elements considered impractical within a regular university course were excluded. The final framework consisted of nine gamification design elements that were considered both pedagogically relevant and operationally feasible for music teacher education.

The selected elements included goals and missions, challenges, feedback, rewards, leaderboards, progress tracking, collaboration, autonomy and choice, and social recognition. Goals and missions provide clear learning objectives and structure learning activities. Challenges introduce appropriate levels of task difficulty and encourage persistence. Feedback supplies learners with information about their performance and supports continuous improvement. Rewards acknowledge achievement and reinforce participation. Leaderboards increase the visibility of progress and achievement. Progress tracking enables learners to monitor their advancement toward learning goals. Collaboration promotes interaction and shared problem-solving among peers. Autonomy and choice support learner agency by providing opportunities to make decisions about learning activities. Social recognition acknowledges contributions and accomplishments within the learning community.

**Figure 3:**
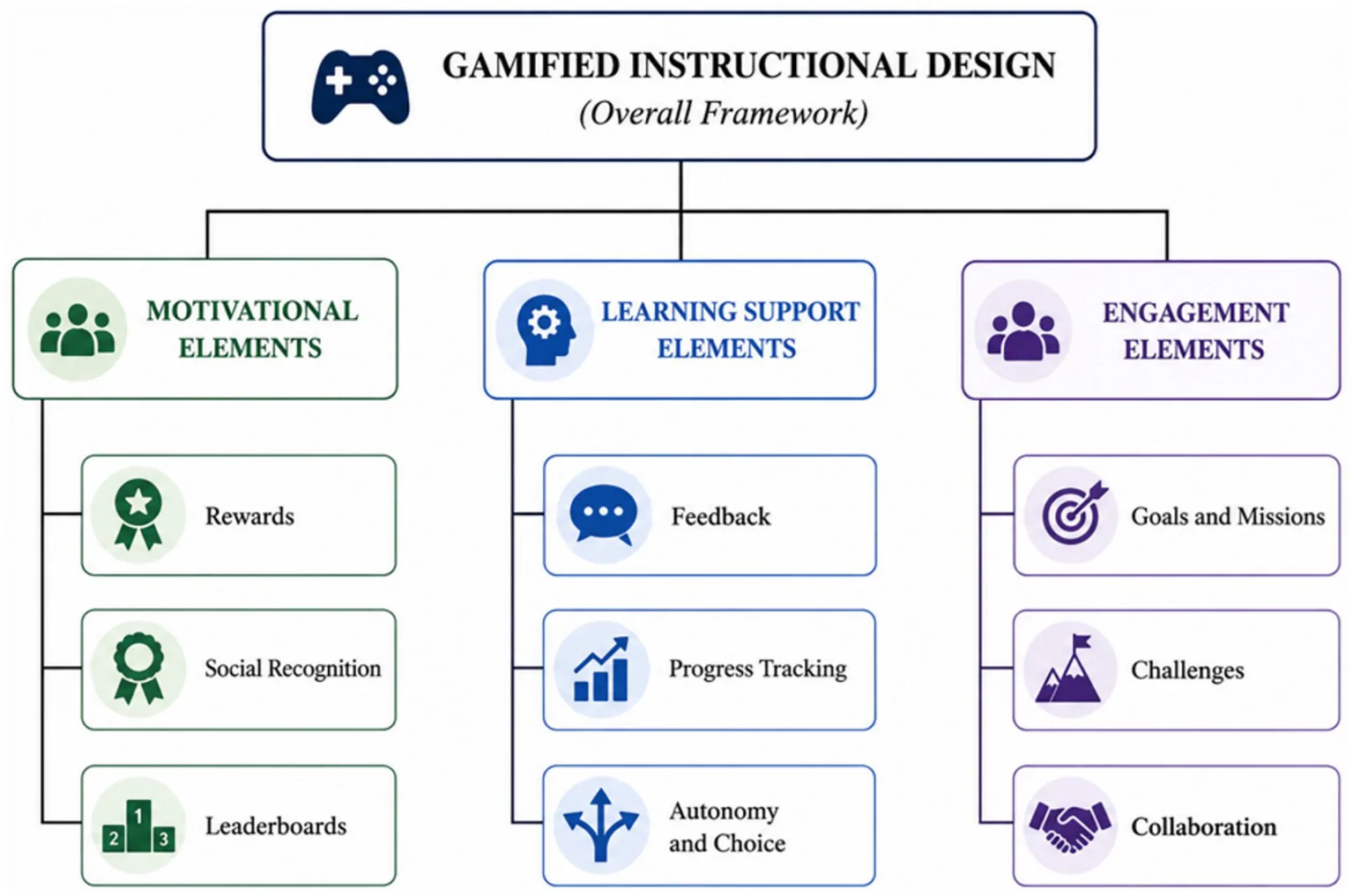
Conceptual framework of the gamification design elements organized into motivational, learning support, and engagement dimensions.

To improve conceptual clarity, these elements were organized into three higher-order dimensions. The motivational dimension comprised rewards, social recognition, and leaderboards. The learning support dimension comprised feedback, progress tracking, and autonomy and choice. The engagement dimension comprised goals and missions, challenges, and collaboration. This hierarchical structure provided the conceptual basis for the DEMATEL analysis and guided the subsequent development of the gamified instructional design. The framework of the selected gamification design elements is presented in Figure 3.

### 2.4 DEMATEL procedure

The Decision-Making Trial and Evaluation Laboratory (DEMATEL) method was employed to determine the relative importance and causal relationships among the gamification design elements presented in Figure 3. DEMATEL is particularly suitable for analyzing complex systems because it identifies both the strength and direction of influence among interrelated factors. In the present study, the method was used to distinguish driving design elements from dependent elements and to establish a structured basis for the subsequent instructional design.

#### 2.4.1 Expert elicitation process

A structured DEMATEL questionnaire was developed based on the nine gamification design elements identified in Section 2.3. The expert panel consisted of 32 higher education teachers with experience in music education, teacher education, curriculum design, educational technology, and gamified learning. Before completing the questionnaire, all experts received standardized instructions regarding the meaning of influence relationships and the evaluation procedure.

Experts assessed the degree to which one design element influenced another using a five-point scale ranging from 0 (no influence) to 4 (very strong influence). Since nine design elements were included in the analysis, each expert completed a 9 × 9 influence matrix with diagonal values fixed at zero. To evaluate the consistency of expert judgments, Kendall’s coefficient of concordance (W) was calculated as follows as Equation 1:

$$W=12S/[m^{2}(n^{3}-n)]$$ (1)

where *S* represents the sum of squared deviations from the mean rank, *m* denotes the number of experts, and *n* denotes the number of evaluated elements. A significant coefficient indicates acceptable agreement among experts and supports aggregating individual judgments. The individual influence matrices were subsequently aggregated using arithmetic means to construct the initial direct-relation matrix as Equation 2:

A=[aij]n×n (2)

where *aᵢⱼ* represents the degree of influence of element *i* on element *j*.

#### 2.4.2 DEMATEL analysis

The DEMATEL analysis followed the standard computational procedure. First, the direct-relation matrix was normalized to eliminate scale effects and ensure comparability among influence values. The normalization coefficient was calculated as Equation 3:

s=1/maxi(∑aij) (3)

The normalized direct-relation matrix was then obtained by Equation 4:

X=sA (4)

where *X* represents the normalized direct-relation matrix.

Next, the total-relation matrix was calculated to capture both direct and indirect influences among the gamification design elements as Equation 5:

$$T=X{\left(I-X\right)}^{-1}$$ (5)

where *I* denotes the identity matrix, and *T* denotes the total-relation matrix.

The total influence dispatched and received by each design element was subsequently calculated. The row sums of the total-relation matrix produced the dispatch influence values as Equation 6:

Di=∑tij (6)

The column sums produced the received influence values as Equation 7:

Ri=∑tji (7)

where *tᵢⱼ* denotes the influence value between elements in the total-relation matrix. Based on these values, two key DEMATEL indicators were computed. The prominence index was calculated as Equation 8:

Di+Ri (8)

This index reflects the overall importance of an element within the system. Larger values indicate stronger involvement in the network of relationships. The relation index was calculated as Equation 9:

Di−Ri (9)

Positive values indicate cause factors that primarily influence other elements, whereas negative values indicate effect factors that are primarily influenced by other elements. To construct the causal network and improve interpretability, a threshold value (*α*) was applied to the total-relation matrix. The threshold was determined using the mean value of all matrix elements as Equation 10:

α=(1/n2)∑∑tij (10)

**Figure 4:**
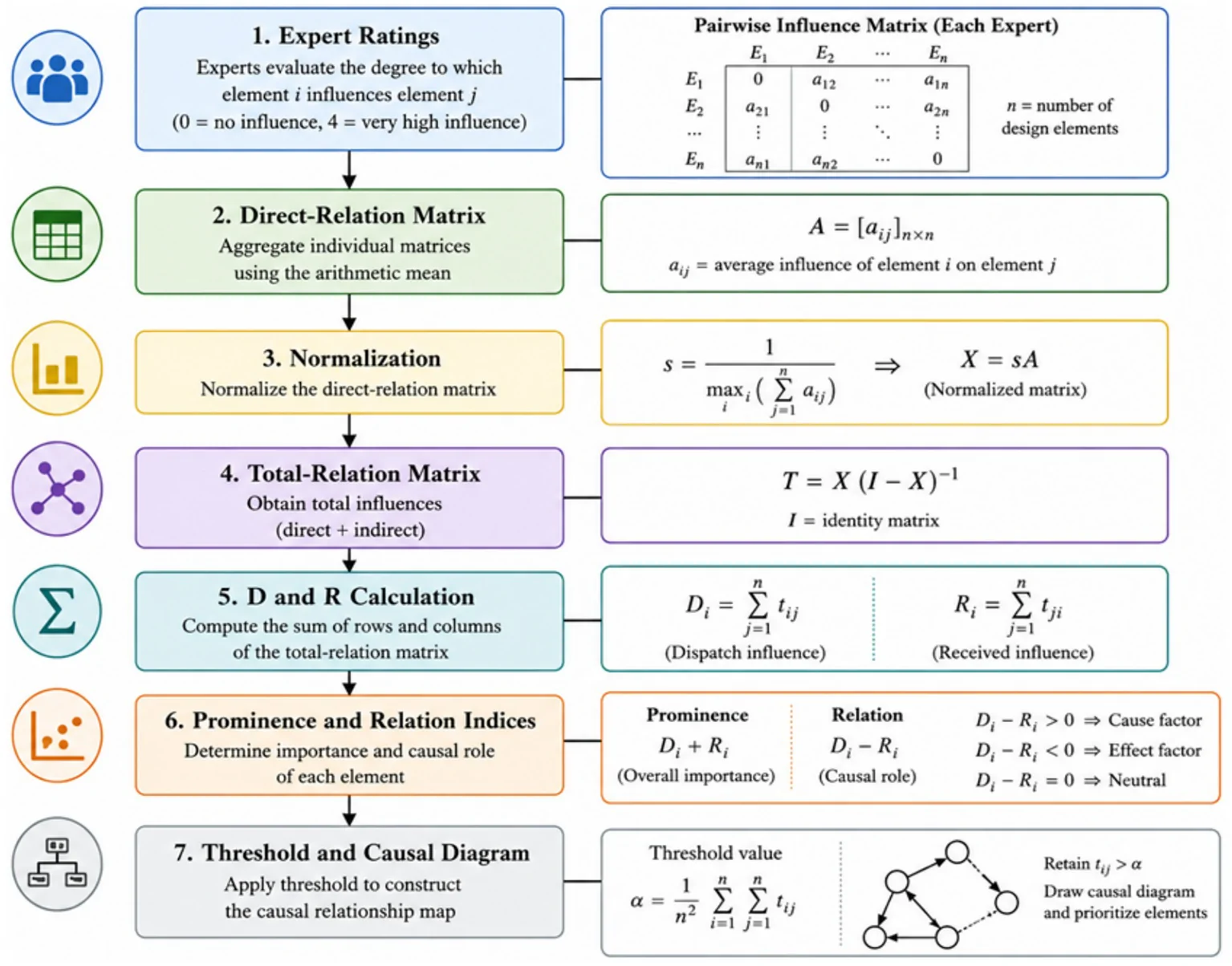
Workflow of the DEMATEL analysis, including matrix construction, normalization, total-relation analysis, prominence and relation calculations, and causal network development.

The threshold value obtained from the total-relation matrix was α = 0.43. Relationships exceeding this value were retained in the causal network, whereas weaker relationships were omitted to improve interpretability. To assess the robustness of the network structure, a sensitivity check was performed using threshold values slightly above and below the selected value (±10%). The resulting cause-and-effect classifications and the ranking of the most prominent gamification elements remained largely unchanged, indicating that the DEMATEL results were stable and not highly sensitive to the threshold selection. Only relationships with values exceeding the threshold were retained in the causal diagram. The resulting prominence and relation indices were used to rank the gamification design elements, identify the most influential factors, and guide the development of the gamified instructional design. The overall analytical procedure is illustrated in Figure 4.

### 2.5 Development of the gamified instructional design

The gamified instructional design was developed based on the prioritized gamification design elements identified through the DEMATEL analysis. The objective of this phase was to translate the analytical findings into a structured learning environment that could support academic engagement and teaching self-efficacy among pre-service music teachers. The design process followed a systematic approach, in which the causal relationships and prominence values from the DEMATEL analysis informed the selection and integration of gamification mechanisms into the course.

The development process consisted of three stages. First, the DEMATEL results were examined to identify the most influential design elements. Elements with high prominence values and positive relation scores were treated as driving factors and were prioritized during instructional planning. Second, these elements were aligned with the learning outcomes, course content, and pedagogical requirements of the music teacher education program. Third, specific gamification mechanisms were embedded into learning activities, assessment tasks, and feedback processes to operationalize the selected design elements.

The resulting instructional design incorporated nine gamification elements organized into motivational, learning support, and engagement dimensions. Motivational elements were implemented through rewards, social recognition, and leaderboards. Learning support elements were incorporated through formative feedback, progress tracking, and opportunities for autonomy and choice. Engagement elements were implemented through goals and missions, progressively challenging learning tasks, and collaborative activities. The integration of these elements was intended to create a balanced learning environment that combined achievement-oriented incentives with opportunities for self-regulation, participation, and social interaction.

**Figure 5:**
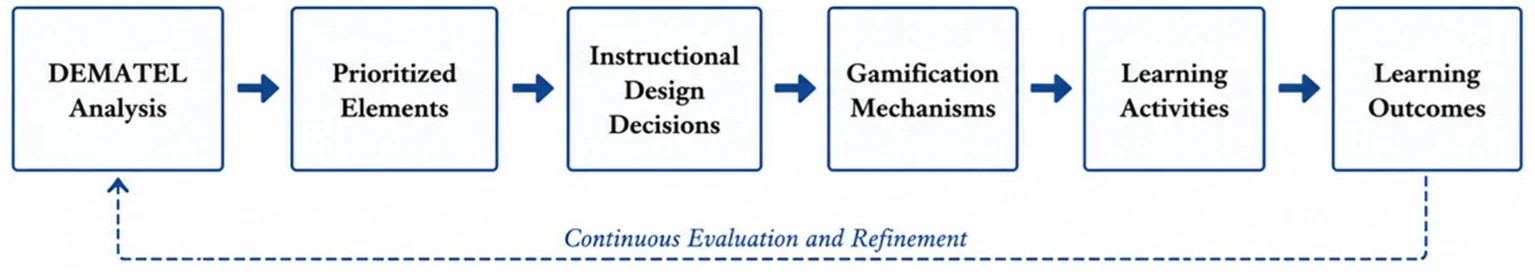
Framework for developing the DEMATEL-informed gamified instructional design from factor prioritization to instructional implementation and learning outcomes.

To ensure pedagogical alignment, all gamification components were mapped to specific learning outcomes and instructional activities. The final design was reviewed by experts in music education and educational technology to evaluate its relevance, feasibility, and consistency with course objectives. Minor revisions were subsequently made before implementation. The overall development process of the DEMATEL-informed gamified instructional design is illustrated in Figure 5.

### 2.6 Intervention procedure

The DEMATEL-informed gamified instructional design was implemented in a music teacher education course during a single academic semester. The intervention was designed to integrate the prioritized gamification elements identified through the DEMATEL analysis into regular teaching and learning activities. The implementation sought to maintain alignment with the course learning outcomes while enhancing student participation, interaction, and engagement throughout the learning process.

#### 2.6.1 Course context and duration

The intervention was conducted within an undergraduate music teacher education course focusing on instructional methods, lesson planning, classroom management, and teaching practice. The implementation lasted 8 weeks and was embedded within the regular course schedule. All participants completed the same learning activities and assessment requirements. Academic engagement and teaching self-efficacy were measured immediately before the intervention and again at the conclusion of the implementation period. The intervention was delivered through the university’s learning management system (LMS) and supported by classroom-based instructional activities. The instructor was responsible for introducing weekly missions, facilitating discussions, providing formative feedback, monitoring progress, and overseeing the implementation of gamification components. Participants were expected to attend scheduled class sessions and complete weekly learning activities, collaborative tasks, reflections, and teaching-related assignments. The estimated student workload was approximately 4–5 h per week, including both in-class and out-of-class learning activities.

#### 2.6.2 Gamification components and implementation

The intervention incorporated the nine gamification design elements identified in the DEMATEL analysis. Goals and missions were used to provide clear learning objectives for each instructional unit. Challenges were incorporated through progressively demanding learning tasks and teaching simulations. Feedback was provided through instructor comments, peer reviews, and formative assessment activities. Rewards were awarded for successful task completion and active participation. Leaderboards were used to display cumulative achievement and participation levels. Progress-tracking mechanisms enabled participants to monitor their progress throughout the course. Collaboration was promoted through group-based learning activities and peer-support tasks. Autonomy and choice were incorporated by allowing participants to select topics, learning resources, or project formats in selected activities. Social recognition was implemented through public acknowledgment of achievement and contribution within the learning environment.

#### 2.6.3 Weekly learning activities

The intervention was organized into eight instructional weeks, with each week incorporating specific learning objectives, instructional activities, and gamification mechanisms. A structured sequence of missions, collaborative tasks, formative assessments, and reflective activities was employed to ensure consistency throughout the implementation period. The weekly instructional plan was designed to progressively develop participants’ pedagogical knowledge and teaching skills while maintaining engagement by integrating gamification elements.

At the beginning of each week, participants received learning objectives and mission requirements. Learning activities included lesson-planning exercises, teaching simulations, classroom-management scenarios, peer discussions, and reflective-practice tasks. Feedback was provided continuously through instructor comments and peer evaluation. Progress indicators, achievement recognition, and collaborative activities were incorporated throughout the intervention period to support sustained participation and learning. The detailed weekly implementation schedule and the corresponding gamification elements are presented in Table 1.

**Table 1:** Weekly implementation schedule of the DEMATEL-informed gamified instructional design and associated gamification elements.

Week	Topic	Learning activity	Gamification elements
1	Course orientation	Mission introduction	Goals, recognition
2	Lesson planning	Collaborative design	Collaboration, feedback
3	Teaching strategies	Challenge activity	Challenges, rewards
4	Classroom management	Case analysis	Feedback, progress tracking
5	Assessment design	Group project	Collaboration, autonomy
6	Music pedagogy	Teaching simulation	Challenges, rewards
7	Microteaching	Peer evaluation	Feedback, recognition
8	Reflection	Final mission	Leaderboards, progress tracking

#### 2.6.4 Implementation Fidelity

Several procedures were employed to support implementation fidelity. A standardized instructional plan was developed before the intervention and was followed throughout the implementation period. The same instructor delivered all learning activities using identical instructional materials and assessment procedures. Gamification components were implemented consistently across instructional sessions in accordance with predefined guidelines. Participation records, activity completion rates, and instructional logs were reviewed periodically to verify adherence to the planned intervention design.

### 2.7 Measures

Two validated instruments were employed to assess academic engagement and teaching self-efficacy. Both instruments were administered at the beginning (pretest) and end (posttest) of the intervention. Responses were recorded on a five-point Likert scale ranging from 1 (strongly disagree) to 5 (strongly agree), with higher scores indicating greater endorsement of the respective construct.

#### 2.7.1 Academic engagement

Academic engagement was measured using the Utrecht Work Engagement Scale for Students (UWES-S). The instrument consists of nine items distributed across three dimensions: vigor, dedication, and absorption. Vigor reflects energy and persistence in learning activities; dedication reflects enthusiasm and commitment to academic tasks; and absorption reflects concentration and immersion in learning experiences. Participants responded to each item using the five-point response scale. An overall academic engagement score was calculated as the mean of all items, with higher scores indicating stronger engagement in course learning activities.

#### 2.7.2 Teaching self-efficacy

Teaching self-efficacy was assessed using the short form of the Teachers’ Sense of Efficacy Scale (TSES). The instrument contains 12 items distributed across three dimensions: instructional strategies, classroom management, and student engagement. Instructional strategies assess confidence in delivering effective instruction, classroom management evaluates confidence in maintaining productive learning environments, and student engagement assesses confidence in motivating and supporting learners. The overall teaching self-efficacy score was calculated as the mean of all items, with higher scores indicating stronger perceptions of teaching capability.

#### 2.7.3 Translation and instrument validation

As the study was conducted in China, all measurement items were administered in Chinese. A translation and back-translation procedure were employed to ensure linguistic equivalence. First, two bilingual researchers independently translated the original English instruments into Chinese. The translated versions were then compared and reconciled into a single version. Subsequently, an independent bilingual translator, who was not involved in the initial translation process, translated the Chinese version back into English. The original and back-translated versions were compared, and minor wording revisions were made to improve semantic equivalence and contextual appropriateness.

Example translated items included “I am enthusiastic about my studies” (Academic Engagement) and “I can effectively implement instructional strategies to support student learning” (Teaching Self-Efficacy). These items were translated into Chinese using the translation and back-translation procedure described above. Where available, previously validated Chinese adaptations of the instruments were consulted during the translation process to enhance linguistic and conceptual equivalence. Minor wording modifications were made to ensure suitability for the music teacher education context.

Prior to the main analysis, the psychometric properties of the instruments were examined. Internal consistency reliability was evaluated using Cronbach’s alpha (*α*) and composite reliability (CR). Construct validity was assessed through confirmatory factor analysis (CFA). Convergent validity was evaluated using average variance extracted (AVE), whereas discriminant validity was assessed by comparing the square root of AVE values with the correlations among latent constructs. Values exceeding the commonly accepted thresholds of α ≥ 0.70, CR ≥ 0.70, and AVE ≥ 0.50 were considered indicative of satisfactory reliability and validity.

The measurement model demonstrated acceptable fit to the data (see Table 2). Model adequacy was evaluated using multiple fit indices, including the chi-square to degrees-of-freedom ratio (χ^2^/df), Comparative Fit Index (CFI), Tucker–Lewis Index (TLI), Root Mean Square Error of Approximation (RMSEA), and Standardized Root Mean Square Residual (SRMR). The resulting reliability and validity statistics are reported in the Results section.

**Table 2:** Reliability and validity statistics of the measurement instruments.

Construct	Items	α	CR	AVE
Academic engagement	9	0.89	0.91	0.58
Teaching self-efficacy	12	0.92	0.93	0.61

### 2.8 Data collection and analysis

Data collection was conducted in two stages: the DEMATEL phase and the intervention evaluation phase. During the first stage, the DEMATEL questionnaire was administered to the expert panel. Participants completed pairwise evaluations of the gamification design elements using the structured influence matrix described in Section 2.4. Completed questionnaires were screened for completeness before aggregation and analysis.

During the second stage, data were collected from the intervention sample using a single-group pretest–posttest design. The pretest was administered during the first week of the course before the implementation of the gamified instructional design. Following the completion of the eight-week intervention, the same instruments were administered as a posttest. Academic engagement and teaching self-efficacy scores were calculated by averaging the corresponding item responses. To ensure confidentiality, each participant was assigned a unique identification code that was used to match pretest and posttest responses while preserving anonymity.

The DEMATEL analysis was conducted according to the procedure described in Section 2.4. Kendall’s coefficient of concordance (W) was calculated to assess the consistency of expert judgments. Subsequently, the direct-relation matrix, the normalized matrix, the total-relation matrix, the prominence values (D + R), and the relation values (D − R) were computed. The resulting indices were used to identify the most influential gamification design elements and their causal relationships.

Prior to the main analyses, the measurement properties of the instruments were examined. Internal consistency reliability was evaluated using Cronbach’s alpha and composite reliability. Construct validity was assessed using confirmatory factor analysis, while convergent and discriminant validity were evaluated using the average variance extracted and inter-construct correlations. Descriptive statistics, including means and standard deviations, were calculated for all study variables.

Pretest–posttest differences in academic engagement and teaching self-efficacy were examined using paired-samples t-tests. Assumptions of normality were evaluated using skewness, kurtosis, and the Shapiro–Wilk test before conducting inferential analyses. Effect sizes were calculated using Cohen’s dz. to assess the magnitude of change. Ninety-five percent confidence intervals were reported for all mean differences. Statistical significance was evaluated at the 0.05 level. All analyses were performed using SPSS 29.0 and AMOS 24.0.

## 3 Numerical results

### 3.1 Measurement model assessment

Prior to examining the DEMATEL results and intervention outcomes, the reliability and validity of the measurement instruments were assessed. Reliability was evaluated using Cronbach’s alpha (*α*) and composite reliability (CR), and convergent validity was assessed using factor loadings and the average variance extracted (AVE). The results are presented in Table 3.

**Table 3:** Reliability and convergent validity statistics of the measurement model.

Construct	Items	Loading Range	α	CR	AVE
Academic engagement	9	0.72–0.86	0.88	0.90	0.56
Teaching self-efficacy	12	0.74–0.89	0.92	0.93	0.61

#### 3.1.1 Reliability and convergent validity

As shown in Table 3, both constructs demonstrated satisfactory internal consistency. The Cronbach’s alpha coefficients ranged from 0.88 to 0.92, substantially exceeding the recommended threshold of 0.70. Similarly, composite reliability values ranged from 0.90 to 0.93, indicating a high degree of consistency among the measurement items. These findings suggest that the instruments yielded stable, reliable measurements of academic engagement and teaching self-efficacy in the study sample.

A comparison of the two constructs revealed that teaching self-efficacy exhibited slightly stronger reliability indicators than academic engagement. Specifically, the teaching self-efficacy scale achieved a Cronbach’s alpha of 0.92 and a composite reliability value of 0.93, compared with corresponding values of 0.88 and 0.90 for academic engagement. Although both constructs demonstrated strong reliability, the higher values observed for teaching self-efficacy suggest greater homogeneity among its measurement items.

The standardized factor loadings ranged from 0.72 to 0.89, indicating that all observed variables contributed meaningfully to their respective latent constructs. None of the factor loadings fell below the commonly accepted threshold of 0.50, suggesting that all retained items adequately represented the intended dimensions. Furthermore, the AVE values of 0.56 and 0.61 exceeded the recommended criterion of 0.50, indicating that the constructs explained more than half of the variance in their indicators. Collectively, these results provide strong evidence of convergent validity and support the adequacy of the measurement mode.

#### 3.1.2 Discriminant validity

Discriminant validity was evaluated using the Fornell–Larcker criterion. The square roots of the AVE values exceeded the corresponding inter-construct correlations, indicating that each construct shared more variance with its own indicators than with other constructs in the model. This finding confirms that academic engagement and teaching self-efficacy represent conceptually distinct dimensions despite their theoretical association within educational contexts. Establishing discriminant validity is particularly important in the present study because both constructs relate to student learning experiences and professional development. The results indicate that the instruments successfully distinguished between participants’ engagement in learning activities and their perceptions of teaching capability, thereby reducing concerns regarding construct overlap or measurement redundancy.

#### 3.1.3 Confirmatory factor analysis

A confirmatory factor analysis was conducted to evaluate the overall fit of the measurement model. The model fit statistics are presented in Table 4. The chi-square to degrees-of-freedom ratio (χ^2^/df) was 2.18, which remained below the recommended threshold of 3.00 and indicated an acceptable level of model parsimony. The Comparative Fit Index (CFI = 0.95) and Tucker–Lewis Index (TLI = 0.94) both exceeded the recommended value of 0.90, indicating that the proposed measurement structure substantially improved over a null model.

**Table 4:** Confirmatory factor analysis results for the measurement model.

Fit Index	Recommended Value	Obtained Value
χ^2^/df	<3.00	2.18
CFI	>0.90	0.95
TLI	>0.90	0.94
RMSEA	<0.08	0.062
SRMR	<0.08	0.051

Additional support for model adequacy was provided by the error-based fit indices. The RMSEA value of 0.062 fell within the range generally considered indicative of a reasonable approximation error, while the SRMR value of 0.051 remained well below the recommended cutoff of 0.08. These results suggest that the discrepancies between the observed and model-implied covariance matrices were relatively small. Taken together, the reliability, validity, and model fit results demonstrate that the measurement model exhibited satisfactory psychometric properties. The findings support the suitability of the academic engagement and teaching self-efficacy instruments for subsequent analyses and provide confidence that the observed relationships among variables are unlikely to be substantially affected by measurement error.

### 3.2 DEMATEL results

The DEMATEL analysis was conducted to identify the relative importance and causal relationships among the nine gamification design elements. The elements were coded as follows: E1 = Goals and Missions, E2 = Challenges, E3 = Feedback, E4 = Rewards, E5 = Leaderboards, E6 = Progress Tracking, E7 = Collaboration, E8 = Autonomy and Choice, and E9 = Social Recognition. The analysis included the assessment of expert agreement, prominence and relation indices, element ranking, and the construction of the causal relationship network.

#### 3.2.1 Expert agreement analysis

Before performing the DEMATEL computations, the consistency of expert judgments was evaluated using Kendall’s coefficient of concordance. The results are presented in Table 5. The obtained Kendall’s W value was 0.71, indicating a substantial level of agreement among the experts. Furthermore, the chi-square test was statistically significant (χ^2^ = 181.47, *p* < 0.001), indicating that the observed agreement was not random. The magnitude of Kendall’s W demonstrates that the participating experts exhibited a relatively consistent understanding of the interrelationships among the gamification design elements. Therefore, the aggregated evaluation matrix was considered sufficiently reliable for the subsequent DEMATEL analysis.

**Table 5:** Expert agreement analysis.

Statistic	Value
Number of experts	32
Kendall’s W	0.71
χ^2^	181.47
df	8
*p*-value	< 0.001

#### 3.2.2 Prominence and relation analysis

The prominence and relation values obtained from the DEMATEL analysis are presented in Table 6. The prominence index (D + R) reflects the overall importance of an element within the system, whereas the relation index (D − R) indicates whether an element functions primarily as a cause factor or an effect factor.

**Table 6:** Prominence and relation analysis of gamification design elements.

Code	Element	D	R	D + R	D − R	Category
E1	Goals and missions	5.38	4.20	9.58	1.18	Cause
E2	Challenges	4.91	4.36	9.27	0.55	Cause
E3	Feedback	5.62	4.32	9.94	1.30	Cause
E4	Rewards	4.11	5.02	9.13	−0.91	Effect
E5	Leaderboards	3.87	4.69	8.56	−0.82	Effect
E6	Progress tracking	5.29	4.83	10.12	0.46	Cause
E7	Collaboration	4.32	4.88	9.20	−0.56	Effect
E8	Autonomy and choice	5.07	4.16	9.23	0.91	Cause
E9	Social recognition	3.95	5.02	8.97	−1.07	Effect

Among all elements, E6 (Progress Tracking) achieved the highest prominence value (10.12), indicating that it occupied the most influential position within the overall network. This finding demonstrates that continuous monitoring of learning progress plays a central role in connecting and reinforcing other gamification mechanisms. E3 (Feedback) ranked second with a prominence value of 9.94, followed by E1 (Goals and Missions) with a value of 9.57. The strong performance of these elements indicates that instructional guidance, performance monitoring, and clearly defined learning objectives constitute the core structure of the gamified learning environment.

The relation values further differentiated the elements into cause-and-effect groups. E1, E2, E3, E6, and E8 produced positive relation values and were therefore classified as cause factors. Among these elements, E1 (Goals and Missions) exhibited the strongest causal influence (D − R = 1.18), indicating that it exerted the greatest influence on other elements within the system. E3 (Feedback) and E8 (Autonomy and Choice) also demonstrated substantial causal effects, confirming their role as key drivers of the instructional design.

In contrast, E4 (Rewards), E5 (Leaderboards), E7 (Collaboration), and E9 (Social Recognition) generated negative relation values and were classified as effect factors. E9 (Social Recognition) recorded the most negative relation value (−1.07), indicating that it was strongly influenced by the other design elements. Similarly, rewards and leaderboards received considerably more influence than they exerted. These findings indicate that recognition-oriented and reward-based mechanisms depend heavily on the successful implementation of the primary instructional drivers.

#### 3.2.3 Ranking of gamification design elements

To facilitate interpretation, the elements were ranked according to their prominence values. The ranking results are presented in Table 7. E6 (Progress Tracking) achieved the highest ranking, followed by E3 (Feedback) and E1 (Goals and Missions). These three elements exhibited prominence values substantially higher than those of the remaining elements, highlighting their central role within the gamification framework.

**Table 7:** Ranking of gamification design elements based on prominence values.

Rank	Code	Element	D + R
1	E6	Progress tracking	10.12
2	E3	Feedback	9.94
3	E1	Goals and missions	9.58
4	E2	Challenges	9.27
5	E8	Autonomy and choice	9.23
6	E7	Collaboration	9.20
7	E4	Rewards	9.13
8	E9	Social recognition	8.97
9	E5	Leaderboards	8.56

The ranking pattern demonstrates that instructional support mechanisms occupied the highest positions in the system. Progress tracking and feedback directly support learner awareness, self-monitoring, and performance improvement, whereas goals and missions provide structure and direction for learning activities. Together, these elements establish the foundation upon which the remaining gamification mechanisms operate.

A second group of influential elements consisted of E2 (Challenges), E8 (Autonomy and Choice), and E7 (Collaboration). Although these elements displayed lower prominence values than the top-ranked factors, they remained strongly connected to the overall system and contributed to learner engagement and participation.

The lowest positions were occupied by E4 (Rewards), E9 (Social Recognition), and E5 (Leaderboards). Despite their importance within gamification environments, the findings indicate that these mechanisms play a more supportive role and derive much of their effectiveness from the instructional and motivational conditions established by the higher-ranked elements.

#### 3.2.4 Causal relationship network

**Figure 6:**
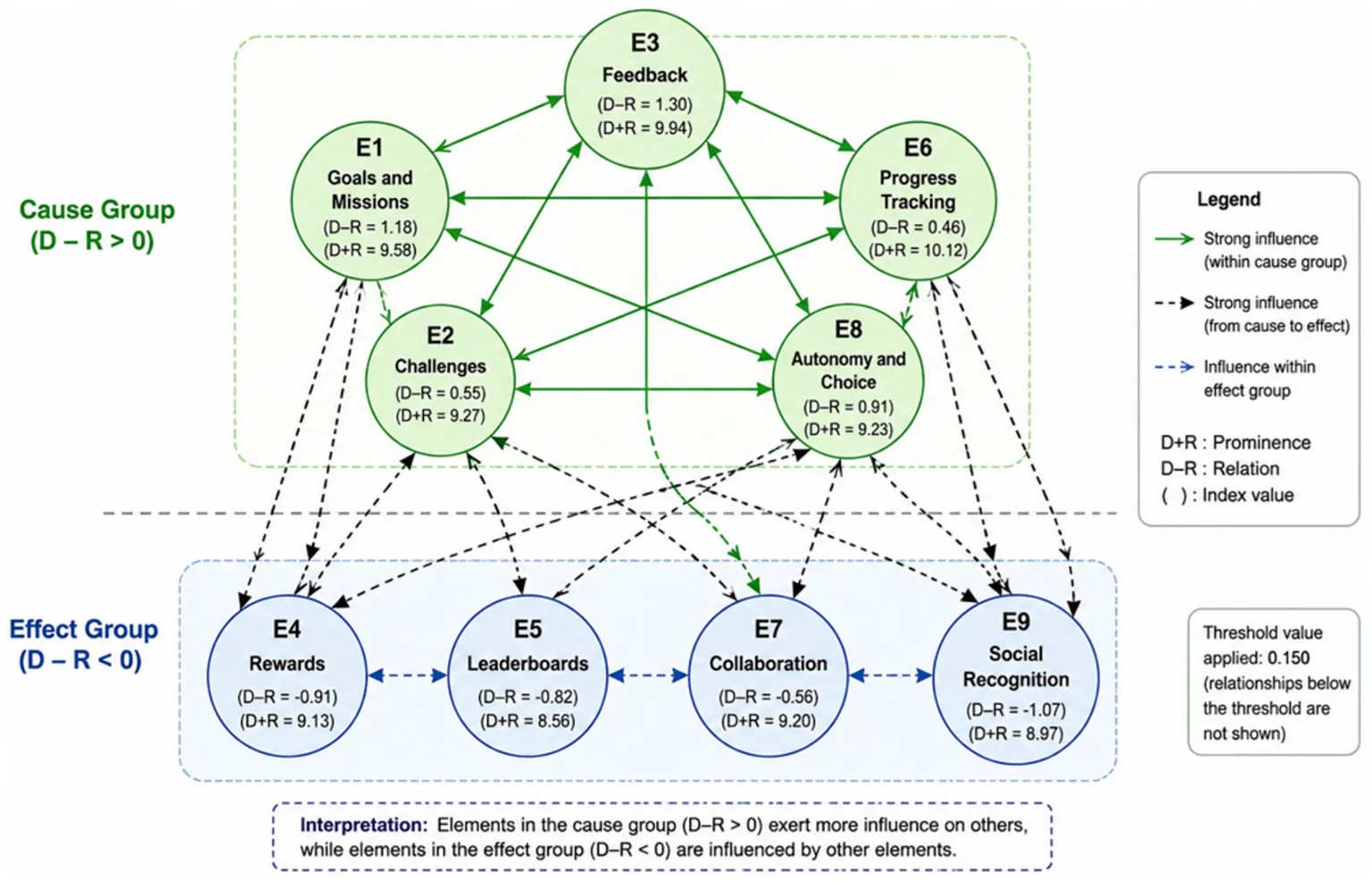
DEMATEL causal relationship network illustrating the prominence and cause–and–effect structure of the gamification design elements (E1–E9).

The causal relationship network derived from the DEMATEL analysis is illustrated in Figure 6. The figure illustrates the interactions among the gamification design elements after applying a threshold to remove weak relationships.

As shown in Figure 6, the cause group consists of E1, E2, E3, E6, and E8. These elements function as the principal driving forces of the gamification framework because they exert greater influence on other elements than they receive. Among them, E3 (Feedback), E1 (Goals and Missions), and E6 (Progress Tracking) occupy the most influential positions due to their high prominence values and positive causal effects. Their central location within the network demonstrates their importance in shaping the overall instructional design.

The effect group includes E4, E5, E7, and E9. These elements are considerably influenced by the cause group and function as dependent mechanisms within the system. Social recognition and rewards are strongly affected by the implementation of feedback, progress monitoring, and goal-oriented learning activities. Consequently, improvements in the primary driving factors are expected to strengthen the effectiveness of the dependent elements. Additional validation of the DEMATEL outputs was achieved through several procedures. First, substantial expert agreement was observed (Kendall’s *W* = 0.71, *p* < 0.001). Second, the prominence and relation indices produced a coherent causal structure that aligned with established theoretical perspectives on gamified learning, particularly the central roles of feedback, progress tracking, and goal-oriented learning activities. Third, the highest-ranked DEMATEL elements were successfully translated into the instructional intervention and demonstrated positive post-intervention changes in the measured outcomes. Although these findings do not constitute causal validation, they provide preliminary support for the practical relevance and applicability of the DEMATEL results.

### 3.3 Descriptive statistics and normality assessment

Descriptive statistics and normality assessments were conducted prior to examining the effects of the DEMATEL-informed gamified instructional design on academic engagement and teaching self-efficacy. The results are presented in Table 8.

**Table 8:** Descriptive statistics and normality assessment of academic engagement and teaching self-efficacy.

Variable	Measurement	Mean	SD	Skewness	Kurtosis	Shapiro–Wilk (p)
Academic engagement	Pretest	3.42	0.58	−0.28	−0.61	0.072
Academic engagement	Posttest	4.11	0.54	−0.74	0.43	0.064
Teaching self-efficacy	Pretest	3.51	0.61	−0.33	−0.47	0.081
Teaching self-efficacy	Posttest	4.19	0.49	−0.69	0.38	0.069

The descriptive statistics indicate that both academic engagement and teaching self-efficacy increased following the intervention. Academic engagement increased from a pretest mean of 3.42 (SD = 0.58) to a posttest mean of 4.11 (SD = 0.54), representing an increase of 0.69 points. Similarly, teaching self-efficacy increased from a pretest mean of 3.51 (SD = 0.61) to a posttest mean of 4.19 (SD = 0.49), representing a gain of 0.68 points.

The standard deviation values remained relatively stable across the two measurement occasions, indicating a consistent distribution of responses among participants. Furthermore, the observed increases in mean scores suggest positive changes in both constructs following participation in the gamified instructional design.

Normality was assessed using skewness, kurtosis, and the Shapiro–Wilk test. The skewness values ranged from −0.74 to −0.28, whereas the kurtosis values ranged from −0.61 to 0.43. These values remained within the commonly accepted range of ±2.00, indicating no substantial departure from normality. Although the Shapiro–Wilk test indicated statistical significance for some variables, the skewness and kurtosis values, together with the sample size, supported the assumption of approximate normality.

### 3.4 Pretest–posttest comparison of academic engagement

A paired-samples *t*-test was conducted to examine changes in academic engagement following the implementation of the DEMATEL-informed gamified instructional design. The results are presented in Table 9.

**Table 9:** Paired-samples *t*-test results for academic engagement before and after the intervention.

Variable	Pretest Mean (SD)	Posttest Mean (SD)	Mean Difference	t	*p*	Cohen’s dz	95% CI
Academic engagement	3.42 (0.58)	4.11 (0.54)	0.69	10.84	<0.001	1.23	[0.56, 0.82]

The findings revealed a statistically significant increase in academic engagement from the pretest phase (*M* = 3.42, SD = 0.58) to the posttest phase (*M* = 4.11, SD = 0.54). The mean difference of 0.69 points indicates a substantial improvement in participants’ engagement levels during the intervention period. The paired-samples t-test confirmed that this increase was statistically significant, *t*(77) = 10.84, *p* < 0.001.

To evaluate the practical significance of the intervention, Cohen’s dz. was calculated. The effect size value of 1.23 indicates a large effect according to conventional interpretation criteria, demonstrating that the observed improvement was not only statistically significant but also educationally meaningful. Furthermore, the 95% confidence interval for the mean difference did not include zero, providing additional evidence of a positive intervention effect.

The magnitude of the increase suggests that integrating DEMATEL-informed gamification elements positively contributed to participants’ involvement in learning activities. The prominence of instructional support mechanisms identified in the DEMATEL analysis, particularly feedback, progress tracking, and goal-oriented learning activities, may explain the observed enhancement in engagement. These elements provide learners with clear learning pathways, continuous performance information, and opportunities for active participation, all of which are closely associated with sustained academic engagement.

The results also indicate a shift from a moderate level of engagement at the beginning of the intervention to a relatively high level of engagement at the end of the implementation period. This pattern supports the effectiveness of the instructional design framework and suggests that the systematic integration of gamification mechanisms can enhance students’ commitment, participation, and involvement in learning activities within music teacher education contexts.

### 3.5 Pretest–posttest comparison of teaching self-efficacy

A paired-samples *t*-test was conducted to evaluate changes in teaching self-efficacy following participation in the DEMATEL-informed gamified instructional design. The results are presented in Table 10. The findings revealed a statistically significant increase in teaching self-efficacy between the pretest and posttest measurements. The mean score increased from 3.51 (SD = 0.61) before the intervention to 4.19 (SD = 0.49) after the intervention, representing a mean improvement of 0.68 points. The paired-samples *t*-test confirmed that the observed difference was statistically significant, *t*(77) = 11.37, *p* < 0.001.

**Table 10:** Paired-samples *t*-test results for teaching self-efficacy before and after the intervention.

Variable	Pretest mean (SD)	Posttest mean (SD)	Mean difference	*t*	*p*	Cohen’s dz	95% CI
Teaching self-efficacy	3.51 (0.61)	4.19 (0.49)	0.68	11.37	<0.001	1.29	[0.56, 0.80]

The magnitude of change was further examined through Cohen’s dz. The effect size value of 1.29 indicates a large practical effect, suggesting that the intervention exerted a substantial influence on participants’ perceptions of their teaching capabilities. Moreover, the 95% confidence interval for the mean difference remained entirely above zero, providing additional evidence for the robustness of the observed improvement.

A comparison of the results presented in Tables 9, 10 indicates that both academic engagement and teaching self-efficacy improved considerably following the intervention. However, the effect size for teaching self-efficacy was slightly greater than that for academic engagement, indicating that the intervention had a stronger influence on participants’ confidence in performing teaching-related tasks than on their overall engagement in learning activities.

The observed improvement in teaching self-efficacy may be attributed to the integration of the high-priority gamification elements identified through the DEMATEL analysis. Elements such as goals and missions, feedback, progress tracking, and autonomy and choice provided participants with continuous opportunities to monitor their performance, receive constructive guidance, and successfully complete progressively challenging instructional tasks. These experiences may have strengthened perceptions of competence and increased confidence in planning, managing, and implementing teaching activities.

Furthermore, collaborative learning activities and social interaction opportunities embedded within the intervention may have contributed to the development of professional confidence through peer support, shared problem-solving, and reflective practice. The combination of instructional guidance and active participation created a learning environment that encouraged both skill development and positive self-perceptions of teaching capability.

### 3.6 Integrated analysis of DEMATEL priorities and intervention outcomes

The integrated findings of the study are summarized in Table 11. The results demonstrate a clear relationship between the priority gamification design elements identified through DEMATEL and the educational outcomes observed during the intervention.

**Table 11:** Integrated summary of DEMATEL findings and intervention outcomes.

Dimension	Key Finding	Evidence	Interpretation
Expert agreement	Strong consensus among experts	Kendall’s *W* = 0.71, *p* < 0.001	Expert evaluations were sufficiently consistent for DEMATEL analysis
Most influential element	E6 (Progress Tracking)	D + R = 10.12	Central driver within the gamification framework
Strongest causal factor	E3 (Feedback)	D − R = 1.30	Major source of influence on other elements
Most dependent element	E9 (Social Recognition)	D − R = −1.07	Highly influenced by other design elements
Academic engagement	Significant improvement	*t* = 10.84, *p* < 0.001, dz. = 1.23	Large positive intervention effect
Teaching self-efficacy	Significant improvement	*t* = 11.37, *p* < 0.001, dz. = 1.29	Large positive intervention effect
Overall outcome	Positive intervention impact	Combined findings	DEMATEL-informed design enhanced learning and professional confidence

The expert agreement analysis confirmed the reliability of the DEMATEL evaluation process. The obtained Kendall’s coefficient of concordance indicated substantial agreement among the experts, supporting the validity of the prioritization procedure. As shown in Table 11, Progress Tracking (E6), Feedback (E3), and Goals and Missions (E1) emerged as the most influential elements within the causal network. These elements achieved the highest prominence values and occupied central positions within the cause group.

A comparison of the cause-and-effect groups revealed that instructional and learning-support mechanisms exerted a greater influence on the overall system than reward-oriented mechanisms. Feedback and progress tracking demonstrated particularly strong causal effects, indicating that learner guidance, performance monitoring, and continuous feedback function as the primary drivers of the gamified instructional design. In contrast, social recognition, rewards, and leaderboards were classified as dependent elements whose effectiveness relied on the successful implementation of the primary drivers.

The intervention outcomes exhibited a pattern consistent with the DEMATEL results. Significant improvements were observed in both academic engagement and teaching self-efficacy following the implementation of the DEMATEL-informed instructional design. The effect size analysis revealed large practical effects for both constructs, with teaching self-efficacy exhibiting a slightly stronger improvement than academic engagement.

The convergence of the DEMATEL findings and the intervention outcomes indicates that prioritizing influential gamification elements contributed to the effectiveness of the instructional design. The central role of feedback, progress tracking, and goal-oriented learning activities appears to have supported both learner engagement and the development of professional confidence. Collectively, these findings demonstrate that the systematic integration of prioritized gamification mechanisms can enhance the effectiveness of music teacher education programs.

## 4 Discussion

The present study examined the use of DEMATEL to identify and prioritize gamification design elements and subsequently employed the resulting framework to develop a gamified instructional design for pre-service music teachers. The findings revealed that the DEMATEL-informed intervention produced substantial improvements in both academic engagement and teaching self-efficacy. Furthermore, the DEMATEL analysis identified progress tracking, feedback, and goals and missions as the most influential elements within the gamification framework. Collectively, these findings demonstrate the value of combining systematic decision-making approaches with gamified instructional design to enhance learning experiences in teacher education.

### 4.1 Interpretation of the DEMATEL findings

One of the most significant findings of the study was the identification of Progress Tracking (E6), Feedback (E3), and Goals and Missions (E1) as the most influential elements within the causal network. These elements achieved the highest prominence values and occupied central positions within the cause group. This result indicates that the effectiveness of gamified learning environments depends primarily on mechanisms that support learner guidance, self-monitoring, and instructional structure rather than solely on reward-oriented features.

The prominence of progress tracking highlights the importance of providing learners with continuous information regarding their advancement toward learning objectives. Progress indicators allow learners to monitor achievements, identify performance gaps, and regulate learning strategies. Within teacher education contexts, such mechanisms are particularly valuable because they encourage reflective practice and support the gradual development of professional competencies.

Similarly, feedback emerged as one of the strongest causal factors within the network. This finding suggests that timely and constructive performance information functions as a key mechanism through which gamification influences learning behaviors. Feedback not only informs learners about their current performance but also guides subsequent actions and supports continuous improvement. Recent evidence has emphasized the critical role of feedback-oriented gamification in sustaining engagement and improving educational outcomes across higher education settings.

Goals and missions also demonstrated substantial causal influence. Clearly defined objectives provide learners with direction and purpose, thereby increasing commitment to learning activities. This finding aligns with previous research indicating that structured challenges and meaningful goals contribute significantly to motivation and persistence in gamified environments.

In contrast, rewards, leaderboards, and social recognition were classified as effect factors. Although these elements remain important components of gamification, the findings indicate that their effectiveness depends on the successful implementation of the primary instructional drivers. This pattern suggests that external rewards alone may be insufficient to produce meaningful educational outcomes unless supported by mechanisms that facilitate learning, reflection, and performance improvement (Li et al., 2023).

### 4.2 Effects on academic engagement

The findings demonstrated a significant increase in academic engagement following the implementation of the DEMATEL-informed gamified instructional design. Participants reported higher levels of engagement after the intervention, and the associated effect size indicated a substantial practical impact.

Several factors may explain this improvement. First, the intervention integrated multiple gamification mechanisms that promoted active participation, continuous feedback, and goal-directed learning. These features encouraged learners to remain involved in instructional activities and provided regular opportunities for interaction and achievement. Second, the prioritization of influential gamification elements through DEMATEL ensured that the intervention focused on mechanisms with the greatest systemic influence rather than relying on isolated or disconnected features.

The findings are consistent with recent meta-analytic evidence demonstrating that gamification positively influences engagement, motivation, and learning outcomes across educational contexts. They are also supported by studies indicating that challenge-based and technology-enhanced gamification environments promote active participation and sustained learning effort.

An additional explanation may be found in self-determination theory. The integration of progress tracking, autonomy and choice, collaboration, and feedback provided opportunities to satisfy learners’ needs for competence, autonomy, and relatedness. Satisfaction of these psychological needs has been associated with stronger engagement and intrinsic motivation in gamified learning environments (David and Weinstein, 2023a; Li L. et al., 2024).

### 4.3 Effects on teaching self-efficacy

The intervention also produced a substantial improvement in teaching self-efficacy. In fact, the observed effect size was slightly larger than that obtained for academic engagement, suggesting that the intervention exerted a particularly strong influence on participants’ perceptions of teaching competence.

This finding is especially important within teacher education because teaching self-efficacy influences instructional decision-making, persistence, classroom management, and professional development. The intervention provided repeated opportunities for participants to engage in lesson planning, teaching simulations, collaborative problem-solving, peer interaction, and reflective practice. These experiences enabled participants to apply pedagogical knowledge in authentic learning situations and receive continuous feedback regarding their performance.

The prominence of feedback and progress tracking within the DEMATEL results offers a plausible explanation for this outcome. Through regular performance information and visible indicators of achievement, participants were able to recognize their development and gain confidence in their teaching capabilities. This process may have strengthened perceptions of competence and increased beliefs regarding their ability to perform teaching-related tasks successfully.

The findings are consistent with previous research conducted within teacher education settings, which reported positive relationships between gamification and professional learning outcomes. They also support recent evidence indicating that teachers respond positively to gamified educational environments when these environments provide meaningful opportunities for achievement, autonomy, and professional growth (David and Weinstein, 2023b; Kaya and Ercag, 2023).

### 4.4 Theoretical contributions

This study contributes to the gamification literature in several ways. First, it extends existing research by integrating DEMATEL with gamified instructional design. While previous studies have primarily examined the effects of individual gamification elements, the present study investigated the causal interrelationships among multiple design elements and identified their relative influence within a unified framework.

Second, the findings provide empirical evidence that instructional support mechanisms exert greater influence on educational outcomes than reward-oriented mechanisms. The identification of progress tracking, feedback, and goals and missions as primary drivers contributes to a more refined understanding of how gamification operates in higher education environments.

Third, the study contributes to teacher education research by demonstrating that gamification can influence not only engagement but also professional beliefs such as teaching self-efficacy. This finding broadens the scope of gamification research beyond academic performance and motivation, highlighting its relevance for professional preparation programs (Cigdem et al., 2024; Lampropoulos and Sidiropoulos, 2024).

### 4.5 Practical implications

The findings provide several practical implications for instructional designers, teacher educators, and higher education institutions. First, the results suggest that the design of gamified learning environments should prioritize feedback, progress tracking, and clearly defined learning goals. These elements demonstrated the strongest influence within the causal network and therefore represent high-priority design considerations.

Second, institutions should avoid excessive reliance on rewards and leaderboards as primary motivational mechanisms. Although these elements contribute to gamified learning environments, the findings indicate that their effectiveness depends largely on the presence of stronger instructional drivers.

Third, teacher education programs may benefit from integrating DEMATEL-informed gamification frameworks into pedagogical training courses. Such approaches can promote learner engagement while simultaneously strengthening teaching self-efficacy and professional confidence.

Finally, the study demonstrates the value of employing systematic decision-making techniques when developing gamified instructional designs. By identifying influential elements before implementation, educators can allocate resources more effectively and create learning environments that maximize educational impact (Saavedra, 2023; Svanberg and Bergh, 2023).

Although gamification often incorporates competitive elements such as leaderboards and public recognition, previous research has shown that competition may generate different responses among learners. While some students experience increased motivation and engagement, others may experience reduced motivation, anxiety, or perceptions of inequity. Consistent with perspectives from Self-Determination Theory and inclusive education, the present instructional design did not rely primarily on competitive mechanisms. Instead, greater emphasis was placed on feedback, progress tracking, collaboration, autonomy, and goal-oriented learning activities, which were identified as the most influential elements in the DEMATEL analysis. Leaderboards and recognition mechanisms were used in a supportive manner and were intended to complement, rather than dominate, the learning experience. Therefore, the findings should not be interpreted as evidence supporting highly competitive gamification environments, but rather as support for a balanced design that combines motivational, instructional, and collaborative elements.

## 5 Conclusion

This study investigated the development and implementation of a DEMATEL-informed gamified instructional design for pre-service music teachers. By integrating the DEMATEL method with gamification principles, the study identified the most influential gamification design elements and subsequently employed these elements to develop a structured instructional intervention. The findings demonstrated that the proposed approach contributed positively to both academic engagement and teaching self-efficacy.

The DEMATEL analysis revealed substantial agreement among the expert panel (Kendall’s *W* = 0.71, *p* < 0.001), supporting the reliability of the expert evaluations. Among the nine gamification design elements, Progress Tracking (E6) achieved the highest prominence value (D + R = 10.12), followed by Feedback (E3) (D + R = 9.94) and Goals and Missions (E1) (D + R = 9.58). Furthermore, Feedback (E3) exhibited the strongest causal influence (D − R = 1.30), while Social Recognition (E9) demonstrated the strongest dependence within the system (D − R = −1.07). These findings indicate that instructional guidance, performance monitoring, and clearly defined learning objectives constitute the primary driving forces within the gamified instructional design.

The intervention findings further demonstrated significant improvements in both outcome variables. Academic engagement increased from a pretest mean of 3.42 (SD = 0.58) to a posttest mean of 4.11 (SD = 0.54), representing a statistically significant improvement, *t*(77) = 10.84, *p* < 0.001, with a large effect size (Cohen’s dz. = 1.23). Similarly, teaching self-efficacy increased from 3.51 (SD = 0.61) to 4.19 (SD = 0.49), *t*(77) = 11.37, *p* < 0.001, with an even larger effect size (Cohen’s dz. = 1.29). These results demonstrate that the intervention generated substantial educational benefits and exerted a particularly strong influence on participants’ confidence in their teaching capabilities.

The study contributes to the literature by extending the application of DEMATEL within educational gamification research and by demonstrating how causal relationships among gamification elements can inform instructional design decisions. Unlike approaches that rely primarily on rewards and competition, the findings highlight the importance of learner-centered mechanisms such as progress tracking, feedback, and goal-oriented learning activities. The study also contributes to teacher education research by providing evidence that gamified instructional environments can strengthen both engagement and professional beliefs related to future teaching practice.

Several limitations should be acknowledged. The study employed a single-group pretest–posttest design without a control group, which limits causal interpretation. The sample was drawn from a single institution and focused exclusively on pre-service music teachers. In addition, the intervention was implemented over a relatively short period, and long-term effects were not examined. The findings should be interpreted considering the study’s single-group pretest-posttest design. Although increases in self-reported academic engagement and teaching self-efficacy were observed following the intervention, causal conclusions cannot be established. Alternative explanations, including maturation, testing effects, novelty effects associated with the gamified learning environment, Hawthorne effects arising from participants’ awareness of being studied, and instructor-related influences, may have contributed to the observed changes. Future research should employ control groups, randomized designs, and longer follow-up periods to strengthen causal inference and examine the sustainability of the reported outcomes.

Future studies should employ experimental and longitudinal designs involving larger samples from multiple institutions and disciplines. Additional investigations may also explore the integration of adaptive learning technologies and artificial intelligence to further optimize DEMATEL-informed gamification frameworks. Despite these limitations, the findings demonstrate that the systematic identification and prioritization of gamification elements can support the development of effective learning environments that enhance academic engagement and teaching self-efficacy among future educators.

## Data Availability

The original contributions presented in the study are included in the article/supplementary material, further inquiries can be directed to the corresponding author/s.
